# Basic Skills in Higher Education: An Analysis of Attributed Importance

**DOI:** 10.3389/fpsyg.2022.752248

**Published:** 2022-02-08

**Authors:** Lourdes Aranda, Esther Mena-Rodríguez, Laura Rubio

**Affiliations:** ^1^Department of Research Methods and Diagnosis in Education, University of Málaga, Málaga, Spain; ^2^Department of Developmental and Educational Psychology, University of Granada, Granada, Spain

**Keywords:** self-perceptions, basic skills, higher education, college students, attributed importance, teaching method

## Abstract

Today, the skills-based approach is increasingly in demand by companies due, in large part, to the fact that it favors the management of human resources by focusing on individual capabilities; which, finally, improves the job profile of a company. As a result, choosing the right candidates has become increasingly selective. Universities, therefore, need to teach skills to improve the incorporation of graduates into the workplace making it as successful as possible. For this reason, it is of special relevance to know if college students consider that the acquisition of skills is key for their incorporation into the workplace. The main objective of this study was to analyze and compare the importance assigned to the acquisition of basic skills in the university education of 694 students studying four different bachelor degrees: pedagogy, early childhood education, primary education, and psychology. For this purpose, a Likert-type questionnaire on basic skills was distributed with four possible options and the following five dimensions that grouped basic skills: organizational and planning capacity; access to information sources; analysis and synthesis of texts, situations, and people; teamwork; and problem solving. The results show that as a whole all students across different bachelor degrees gave a high score to the acquisition of basic skills, with early childhood education students giving it greater importance compared to the students from other disciplines and, more specifically, differences were observed in some dimensions depending on the bachelor degree that they have started.

## Introduction

In recent decades, a continuous and profound change has been taking place in social and labor reality, necessitating universities to regularly adapt to the new professional needs that the labor market demands from future graduates (Martínez-Clares and González-Morga, 2018; Pineda-Herrero et al., 2018; Rodríguez-Gómez et al., 2018). In fact, it is well known that the prestige of universities depends on the success of graduates, and this is one of the pillars of the triple helix, along with industry and government (Etzkowitz and Leydesdorff, 2000).

With the old university model, educational institutions sought to prepare professionals without paying much attention to the labor insertion of these people once they had completed their university studies, and without concern about whether they would be able to acquire a job associated with their qualification. However, in Spain, BOE (1983), resulted in the implementation of a new training model based on skills, and university education has undergone an important transformation which has generated a new university model in which a great interest can now be seen in all aspects concerning the insertion in the job market of professionals trained at universities (Eslava-Suanes et al., 2018; García-Blanco and Cárdenas, 2018; Gutiérrez et al., 2019; Baquero and Ruesga, 2020). According to Torres and Vidal (2015), “To talk about employability is to talk about aptitudes and attitudes, about a vital syllabus and about good personal qualities; that is, better or worse possibilities of access and adaptation to the job world” (p. 65).

If not addressed, the expense that this education entails would represent a relevant social and economic “loss,” since the money and resources spent on training these students would not be economically or socially profitable, nor would there be any benefit from the time and resources used (Galcerán, 2010, p. 94).

One of the key aspects in this transformation of the teaching–learning processes in European universities, and specifically Spanish ones, is the syllabus design based on learning by skills (Pozo and Bretones, 2015; Calderón et al., 2018). However, even though more than two decades have passed since the implementation of a syllabus based on the acquisition of skills in European universities, the member states point out a multitude of difficulties in implementing this methodology (Ramírez-García, 2016).

In a more general sense, the term “skills” refers to the norms, techniques, procedures, attitudes, and abilities that future graduates acquire as they go through university, to perform their professional functions appropriately (Biccoca, 2018; Martínez-Clares and González-Morga, 2019; Gargallo et al., 2020). Therefore, the emphasis on skills “would mark the importance given to the student’s own learning and to the development of their ability to interact creatively with the environment” (Galcerán, 2010, p. 104).

In the Tuning Educational Structures in Europe Project, an exhaustive distinction of skills or competences is presented: “[…] skills can be divided into skills related to a field of expertise (specific to a field of study), and generic skills (common for different courses)” (Tuning Project, 2007, p. 37). The generic or basic skills identify shared attributes which could be general to any degree, such as the capacity to learn, decision making capacity, project design and management skills, which are developed in all study programs. On the other hand, subject-specific skills refer to theoretical, practical and experimental skills and knowledge for a specific area or study program. Therefore, it can be said that transversal, generic, or basic skills refer to the elements common to any degree, while specific skills refer to the elements of each degree. In Spain, Ministry of Education and Science (2007) by which the organization of official university education was established, reference was already made to the establishment of two types of skills in college education with the purpose that the knowledge, skills, and abilities acquired in universities can be adequately adapted to the demands of the job market.

Nevertheless, there is still a significant gap between the knowledge, skills, and abilities that students acquire in their college studies and the required demands of the job market (Planas, 2010; Wild and Schulze-Heuling, 2020). Following this line, some authors point to the existence of a clear mismatch between the capacities and abilities learned by students in universities and the professional capacities required in the labor market for a successful performance of the tasks and functions in different jobs (Martínez-Clares and González-Morga, 2018; Aranda et al., 2021). This mismatch is currently presented as a great problem in the higher education system and, therefore, it is recommended that all the agents that make up the educational system reach consensus on the practical training of students (Pujol-Jover et al., 2015; Cabezas-González et al., 2017).

It is recommended that, at all levels in educational institutions, the development and acquisition of skills is promoted, and especially of transversal, generic, or basic skills, which are those that really provide the student body with great flexibility in different work functions and the ability to adapt to different jobs (Méndez et al., 2015; Rodríguez-Gómez, 2018). In this sense, there are various works that are relevant to the role of praxis by teaching staff, since they consider that professors are really those who are trained to improve and innovate the learning of college students (Biesta, 2015; La Rosa, 2015; Méndez et al., 2015).

Less importance has been given to the figure of the student body. However, college students must be motivated to acquire skills. Previous research shows that, even in stages prior to university, student interest is a key factor for adequate academic training (Rodríguez et al., 2020). All of this, together with the increasingly active role of the student body in their training (Aranda et al., 2015; López-Núñez et al., 2019), renders the attitude and motivation of students in university education with regard to skills as relevant (Centeno and Cubo, 2013).

Previous research analyses the perception of students in the acquisition of competences in the different university degrees (Gimeno-Santos and Martín-Cabello, 2007; Gómez-Puertas et al., 2014; Belmonte-Almagro et al., 2019). It is worth highlighting the work carried out by Belmonte-Almagro et al. (2019), where it is shown how, at a global level, students value the acquisition of competences in universities acceptably. Following this same line of research, in the study by Gimeno-Santos and Martín-Cabello (2007), a high score was also obtained in the importance attributed to the acquisition of competences in the Degree of Psychology by the students. More specifically, in the work of Gómez-Puertas et al. (2014), it is concluded that the students of the Degree in Journalism manifest a positive assessment in the acquisition of skills related to the capacity for critical and reflective analysis on their own actions.

The fact that students value the acquisition of competences very positively is of special relevance, since, if university students do not value training through the acquisition of competences, they will not be motivated and, therefore, they will not be adequately trained professionally. It is important that students value skills favorably, since they are an active part of the teaching-learning process.

Therefore, the general objective of this research was to analyze the importance assigned to the acquisition of generic or basic skills by college students in 1st-year study of different degrees and to identify if there are differences in said assessment depending on the degree under study. This research attempted to answer the following questions: What assessment do students give to the acquisition of skills in higher education when they begin their undergraduate studies? Are there differences among the students regarding the importance attributed to the acquisition of skills depending on the degree they are studying?

## Materials and Methods

### Participants

The sample was selected through a non-probabilistic and intentional sampling, based on accessibility criteria. This sample consisted of 694 students belonging to four disciplines in the faculties of Educational Sciences and Psychology at the Universities of Malaga, Granada, and Seville, specifically in pedagogy, early childhood education, primary education, and psychology (see Table 1). Of the total participants, 6.9% were men and 93.1% were women. The difference in ratio between male and female students is due to the fact that the degrees considered in this research are biased by gender. As is well known, the educational trajectories between women and men differ and in some subjects one gender prevails over another. Specifically, in the degrees considered in this study, the female gender predominates over males.

**TABLE 1:** Distribution of students by bachelor degree.

Degree	*n*	Percentage
Pedagogy	133	19.2%
Early childhood education	183	26.3%
Primary education	279	40.2%
Psychology	99	14.3%
Total	694	100%

### Instruments

To assess the importance given to basic skills, a questionnaire consisting of 52 items was used, adapted from Gimeno-Santos and Martín-Cabello (2007). The response format used was a Likert 4-point scale with the following options (1 = not at all, 2 = little, 3 = fairly, and 4 = a lot).

The items were grouped into five dimensions, each related to the basic skills expected of college students: Organizational and planning capacity (OP; e.g., “Have the necessary information to be able to carry out an academic work”); access to information sources (AF; e.g., “Knowing how to search in a library or newspaper archive for all the information you need”); analysis and synthesis of texts, situations, and people (AT; e.g., “Be able to synthesize a text”); teamwork (TE; e.g., “Actively listen to people”); and problem solving (RP; e.g., “Ability to understand that the same situation can have different ways of solution”).

The reliability of the questionnaire was calculated using Cronbach’s Alpha test based on the analysis of the responses of the 694 male and female students surveyed, and shows a high index of internal consistency (α = 0.895). For each scale, the reliability values were also adequate; specifically, for organizational and planning capacity, the α value was 0.716; access to information sources, 0.849; analysis and synthesis of texts, situations, and people, 0.835; teamwork was 0.725; and finally, in the problem-solving dimension, the α value was 0.800.

### Procedure

All data were collected during the second term of the academic year 2018–2019 and 2019–2020 at the Universities of Málaga, Granada, and Sevilla. Table 2 shows the distribution of students by universities and degrees.

**TABLE 2:** Distribution of students by universities and degrees.

University	Degree	*n*
University of Málaga	Pedagogy	133
	Early childhood education	183
	Primary education	54
	Psychology	55
University of Granada	Primary education	117
	Psychology	44
University of Sevilla	Primary education	108
Total		694

At the beginning of each data collection session, a brief explanation of the test was given to the participants, urging them to answer the question “if you were a professional of… do you regard it as important.” In addition, informed consent was requested and provided, and the confidential nature of the information collected was communicated.

### Data Analysis

The methodology used in this research was quantitative. First, a descriptive analysis of the variables among the degree courses and the different dimensions contemplated in the questionnaire was conducted. Subsequently, a *t*-test analysis to assess gender differences, and an analysis of variance (ANOVA) test was run to determine if there were differences in the importance given to skills based on the course taken. Then, in order to verify the differences between the groups, Tukey’s *post-hoc* test (multiple comparisons) was performed. Finally, to estimate the effect size, Cohen’s d for *t*-test and partial eta square (η^2^_*p*_) for ANOVA was applied, with the following considerations in terms of value: for Cohen’s d values of 0.1 represents a small effect size, 0.3 represents a medium effect size and 0.5 represents a large effect size (Field et al., 2012); for partial eta square: 0.0099 = small, 0.0588 = medium, and 0.1379 = large (Richardson, 2011).

## Results

**FIGURE 1:**
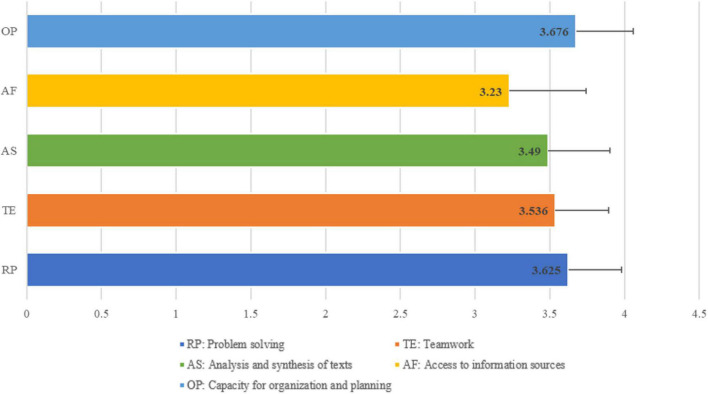
Mean scores of basics skills.

First, the means and standard deviations by dimension were analyzed, and the findings were that students, in general, attribute a high or very high average score to the importance of basic skills, with values exceeding three points out of four (see Figure 1). Therefore, it can be said that the skills perceived by students as more important or necessary for their professional development would be those framed in dimension 1 (organizational and planning capacity) followed by dimension 5 (problem solving), while those perceived as the least important for their professional career would be in dimension 2 (access to information sources).

Regarding gender, significant differences were observed between men and women in the importance given to Capacity for organization and planning (*t*_(687)_ = −3.675, *p* < 0.001, *d* = 0.374) and Problem solving (*t*_(687)_ = −3.476, *p* = 0.001, *d* = 0.354). In both cases, women scored higher than men in the importance given to these basic skills. In the case of Capacity for organization and planning, women scored on average 3.701 (SD = 0.375) and men 3.55 (SD = 0.385), for Problem solving women’s scores were on average 3.647 (SD = 0.350) and for men 3.521 (SD = 0.359).

Subsequently, an ANOVA was carried out to explore whether there were differences in the importance given to basic skills according to university degree (Table 3). The results in all the analyses were significant, so the degree taken influences the importance attributed to the skills. The effect sizes were in all cases medium, with the lowest being for teamwork (0.039) and the highest for organization and planning capacity (0.070) and problem solving (0.066).

**TABLE 3:** One-way Analysis of Variance (ANOVA).

Basic skill	Degree	*M* (SD)	*F*	*p*	η*^2^_*p*_*
OP	Pedagogy	3.59 (0.45)	17.186	0.000	0.070
	Early childhood education	3.84 (0.21)			
	Primary education	3.61 (0.40)			
	Psychology	3.65 (0.37)			
AF	Pedagogy	3.28 (0.46)	12.650	0.000	0.052
	Early childhood education	3.32 (0.42)			
	Primary education	3.07 (0.50)			
	Psychology	3.25 (0.51)			
AT	Pedagogy	3.49 (0.41)	10.105	0.000	0.042
	Early childhood education	3.58 (0.32)			
	Primary education	3.37 (0.40)			
	Psychology	3.44 (0.45)			
TE	Pedagogy	3.54 (0.38)	9.334	0.000	0.039
	Early childhood education	3.64 (0.28)			
	Primary education	3.47 (0.35)			
	Psychology	3.49 (0.41)			
RP	Pedagogy	3.59 (0.40)	16.251	0.000	0.066
	Early childhood education	3.77 (0.24)			
	Primary education	3.56 (0.35)			
	Psychology	3.54 (0.39)			

*OP: capacity for organization and planning; AF: access to information sources; AT: analysis and synthesis of texts, situations, and people; TE: teamwork; RP: problem solving.*

To determine the differences between groups, the Tukey *post-hoc* contrast test was performed. The mean scores for each dimension by bachelor degree are shown in Table 3.

With reference to the first dimension analyzed (capacity for organization and planning), significant differences are observed between the importance given to this dimension by the early childhood education degree students, whose score is significantly higher than students in the pedagogy degree (*p* < 0.001; 95% CI = [0.1446, 0.3603]), primary education degree (*p* < 0.001; 95% CI = 0.1333, 0.3149]) and psychology degree (*p* < 0.001; 95% CI = [0.0696, 0.2980]).

An analysis of the second dimension (access to information sources), shows that primary education degree students score significantly lower in this dimension compared to students in pedagogy (*p* < 0.001; 95% CI = [−0.3431, −0.0814]), early childhood education (*p* < 0.001; 95% CI = [−0.3668, −0.1302]), and psychology (*p* < 0.01; 95% CI = [−0.3246, −0.0452]) degrees.

In the case of the third dimension (analysis and synthesis of texts, situations, and people), the Tukey test revealed differences in the scores of studying early childhood education degree compared to primary education (*p* < 0.001; 95% CI = [0.1133, 0.3088]) and psychology (*p* < 0.05; 95% CI = [0.0185, 0.2645]) degrees, with early childhood education degree students scoring the highest in this dimension. In addition, students of the degree in pedagogy scored higher than those in primary education degree (*p* < 0.05; 95% CI = [0.129, 0.2291]).

Referring to the analyses carried out on the fourth dimension (teamwork), statistically significant differences are once again observed between students of the pedagogy and early childhood education degrees (*p* < 0.05; 95% CI = [−0.2104, −0.0031]), and between the early childhood education degree students and those taking the primary education (*p* < 0.001; 95% CI = [0.0855, 0.2599]) and psychology degrees (*p* < 0.01; 95% CI = [0.0456, 0.2651]), respectively. In all cases, the group of early childhood education degree students attributed greater importance to the teamwork dimension.

Finally, if we consider dimension 5 (problem solving), again the students of the early childhood education degree obtain a significantly higher score when compared with the students of the pedagogy (*p* < 0.01; 95% CI = [0.0794, 0.2815]), primary education (*p* < 0.001; 95% CI = [0.1211, 0.2912]) or psychology (*p* < 0.001; 95% CI = [0.1226, 0.3366]) degrees.

In short, there are statistically significant differences between all the degrees participating in this research with slight nuances although, after the analyses, the students in early childhood education degree stand out as the group that placed the highest values on the five dimensions measured by the questionnaire, as basic and necessary skills required by an educational professional.

## Discussion

The objective of this paper was to evaluate the importance assigned to the acquisition of skills by 1st-year college students of different degrees and to identify if there are differences in such assessment depending on the type of studies, since different studies (Rabanal et al., 2020; Sarceda and Barreira, 2021) show that academic training in competencies is one of the key points in the face of labor insertion, a comprehensive training that considers both personal and social motivations so highly valued in the world of work.

Overall, the results obtained show high scores in the importance attributed to basic skills by the four degrees considered in this research. Although a positive assessment by the students of the different groups is observed, according to our results, on the one hand, statistically significant differences were observed between the different study groups (degrees) and by gender, although with a moderate effect size. The sample of this work had a greater number of female participants, something common in education and psychology degrees in Spain. The two skills that have shown significant differences are usually associated with males, since there are gender stereotypes in relation to educational skills such as problem solving (Zhu, 2007). It is possible that women, considering that they possess these skills to a lesser extent, attach more importance to their development than men.

On the other hand, we note that the early childhood education students placed highest value on the five basic skills included in this research compared to the three remaining groups (pedagogy, primary education, and psychology). All these differences observed between the different degrees can be explained, at least in part, by the characteristics of each degree and the target audience they are targeting.

Following this line of thought, the study undertaken by Meroño et al. (2018) indicates that, in addition to the perception of teachers and educational agents, it is necessary to be aware of the perception of students in their own skills learning since their opinion is essential to improve the learning processes in terms of skills. This is especially important in students with special educational needs, since the development of their abilities and skills require specialized attention from teachers (Tanu and Kakkar, 2018; Kakkar, 2020).

This aspect arouses great interest, since the students’ perceptions of their own knowledge, the importance they give to the teaching methodology, and the motivation toward their teaching process is key to achieving greater involvement in their own training (Castells et al., 2015; Martínez-Clares and González-Morga, 2018). In fact, one of the great challenges of the university is that the students become the main figure in their entire college learning process by actively participating in their training (Silva, 2017; Pegalajar, 2020).

The importance of this research has to do with a recently coined term, “academic commitment,” which could be defined as a concept that includes a wide variety of student behaviors and academic practices such as time spent on academic tasks, adaptability, social and academic integration, and teaching methodology (Kahu and Nelson, 2018). Basically, this concept refers to the importance of the opinions and well-being of the students in every way in order to achieve adequate academic preparation that helps them face the important changes that are taking place in society and specifically the demands of the job market (Martínez-Clares and González-Lorente, 2018).

Therefore, it becomes necessary to remove one of the major drawbacks to planning and developing teaching–learning methodologies taught in universities such as the importance that has always been ascribed to the theoretical aspect of the subjects compared to the practical function. Several authors have pointed out that, in general, at university much importance is given to the theoretical content of subjects, while the job market demands that future workers “know how to do it” (Alonso et al., 2009; Jackson, 2012; Torres and Vidal, 2015). This explains why some authors demand greater coordination between university training and the demands of the job market (Cabrera et al., 2016).

Similarly, Ellwanger and Andreucci (2017) refer to the need for college professor teachers to undertake comprehensive training of students, so that in addition to theoretical knowledge, students develop practical and motivational skills. Regarding the development of practical skills, an important issue must be kept in mind, that is, to efficiently implement skills in the current academic curriculum design in universities (Calderón et al., 2018; Glaesser, 2019; Ahmed and Khairy, 2020). This inevitably leads us to reflect on the training of professors in higher education to apply teaching–learning strategies based on skills, particularly basic skills. At this point, several problems in universities can be highlighted, the main issue being that despite working in a higher-level institution the vast majority of university teaching staff have not had specific training outside of the skills of their field of expertise, let alone received pedagogical training to carry out their professional careers, unlike the other educational levels. This is a paradox since they are required to teach skills without having previously received any training in this regard. Faced with this professional challenge, the pedagogical training of professors is key to professional success (Más-Torello, 2011).

Another problem faced by professors is that they generally have very high ratios in the number of male and female students, and this renders a more personalized teaching among professor-students that favors the acquisition of skills impossible. Furthermore, the time availability of college teaching staff must be considered since, very often, professors do not have the available time to enable them to propose subjects, including the skills to be developed and the way to assess them (Villarroel and Bruna, 2014). For this reason, it is essential that teachers participate in and grant special dedication to the inclusion of basic skills in their methodological strategies (Villarroel and Bruna, 2014). It is essential for professors to reflect on and review these methodological strategies when considering the perception and importance placed by students on the acquisition of transversal, generic, or basic skills in their college studies (Rodríguez-Gómez et al., 2018), as it has been possible to verify after the results of this research, since the 5 groups of students have a great motivation toward learning by competences.

Based on the above, we can affirm that, if students consider the inclusion of skills in the syllabus to be important, they will show a predisposition to be part of their skills learning and, with this, progress can be made in two basic aspects in the higher educational context. One of these aspects would be the progressive increase in the participation of students in their own college education, thus achieving a more autonomous and active role (López-Núñez et al., 2019); and the other would be motivation in teaching methodologies, which is key to performance and would lead to academic success.

Within the inclusion of skills in the syllabus of the different degrees of our universities, the so-called generic, transversal, or basic skills are of special interest since, if the development of these most basic abilities and skills is encouraged, students will learn to adapt more satisfactorily in the social sphere and, more specifically, in the workplace in increasingly changing contexts. In this sense, Brussels has proposed the preparation of students for their adequate adaptation to the increasingly profound changes in the job market, training citizens of increasingly digital and global societies (Consejo de la Unión Europea, 2018).

This study has some limitations: Although the sample is large, the data was collected at a single point in time. In the future, longitudinal studies should be added to establish the importance given to skills changes as students advance through the years. On the other hand, it would be useful to know the importance ascribed to these skills by those studying college degrees in other fields and to compare the results.

## Conclusion

In conclusion, universities must prepare both professors and their students for the new challenges of the 21st century. It is necessary for professors to train in skills to be able to teach them. In addition, it is important to take into account the attitude and perception of all educational agents, and more especially the perception of students as the main educational agent in the university context. The need to identify which skills are demanded by the job market to adjust both the basic and specific skills of students in an academic context to the labor demands must be considered. For this reason, it is also essential to pay attention to the companies that employ graduates, since they provide hints on the skills currently being demanded in a changing society in which employment has to gradually transform and reinvent itself on a daily basis. In this way, the educational quality will increase, and the professional success of the students will be more likely.

## Data Availability Statement

The raw data supporting the conclusions of this article will be made available by the authors, without undue reservation.

## Author Contributions

LA designed the study, supervised the data collection, and wrote the manuscript. EM-R carried out the statistical analysis and wrote the manuscript. LR supervised the data collection and assisted with writing the article and edited the manuscript. All authors contributed to the article and approved the submitted version.

## Conflict of Interest

The authors declare that the research was conducted in the absence of any commercial or financial relationships that could be construed as a potential conflict of interest.

## Publisher’s Note

All claims expressed in this article are solely those of the authors and do not necessarily represent those of their affiliated organizations, or those of the publisher, the editors and the reviewers. Any product that may be evaluated in this article, or claim that may be made by its manufacturer, is not guaranteed or endorsed by the publisher.
